# Biovalorization of Market Surplus Bread for Development of Probiotic-Fermented Potential Functional Beverages

**DOI:** 10.3390/foods11030250

**Published:** 2022-01-18

**Authors:** Thuy-Linh Nguyen, Mingzhan Toh, Yuyun Lu, Sebastian Ku, Shao-Quan Liu

**Affiliations:** 1 Department of Food Science and Technology, Science Drive 2, Faculty of Science, National University of Singapore, Singapore 117542, Singapore; linhnguyen14@outlook.com (T.-L.N.); toh.mingzhan@gmail.com (M.T.); 2 Lesaffre Singapore Pte. Ltd., 23A Serangoon North Ave 5, #04-09, Singapore 554369, Singapore; s.ku@lesaffre.com; 3 National University of Singapore (Suzhou) Research Institute, 377 Lin Quan Street, Suzhou Industrial Park, Suzhou 215123, China

**Keywords:** bread, biovalorization, probiotics, *Lacticaseibacillus rhamnosus*, *Saccharomyces cerevisiae*, functional beverages

## Abstract

Bread wastage is a growing concern in many developed countries. This research aimed to explore the biovalorization of market surplus bread for the development of probiotic-fermented beverages in a zero-waste approach. Bread slurries with different initial total solid contents were inoculated with probiotics *Lacticaseibacillus rhamnosus* GG (LGG) and *Saccharomyces cerevisiae* CNCM I-3856, alone and in combination. Our results showed that, of all percentages tested, 5% (*w*/*w*, dry weight) initial total solid content resulted in better growth of the probiotics and higher cell counts, while the texture of bread slurries with concentrations higher than 5.0% was too thick and viscous for bread beverage developments. In addition, the development of probiotic-fermented bread beverages was feasible on various types of bread. Furthermore, food additives (sweetener and stabilizer) did not affect the growth of LGG and *S. cerevisiae* CNCM I-3856 in both mono- and co-culture fermentation. During shelf life measurement, co-inoculation of LGG with *S. cerevisiae* CNCM I-3856 significantly improved the survival of LGG compared to the mono-culture at 5 and 30 °C, demonstrating the protective effects provided by the yeast. Our study suggests the potential of using market surplus bread as raw materials to deliver live probiotics with sufficient cell counts.

## 1. Introduction

Probiotics are referred to as “live microorganisms which upon ingestion in 10^8^–10^9^ colony forming units (CFU) per serving exert health benefits on its host beyond inherent general nutrition” [1]. It is believed that the health benefits delivered by probiotics are mainly due to their ability to colonize the gastrointestinal tract, contributing to the establishment of a healthy and balanced intestinal microflora [2]. In addition to probiotic effects, consumption of probiotic-fermented foods can also deliver paraprobiotic (dead probiotic cells) and postbiotic (probiotic metabolites) benefits, as well. 

In general, health benefits delivered by probiotics, paraprobiotics (non-viable cells) [3] or postbiotics (bioactive cellular components and metabolites) [3,4] include increases in lactose tolerance, improvement of intestinal microbiota, increases in antioxidant, anti-inflammatory, immunomodulatory, anti-obesogenic, antihypertensive, and antiproliferative activities [2,3]. In recent years, probiotic foods, especially probiotic beverages, have been receiving increasing interest. Most probiotic beverages are fermented dairy drinks with lactic acid bacteria (LAB) [4], as milk proteins have the buffering capacity that supports the viability of the bacteria in an acidic medium. The major drawbacks of probiotic dairy products are consumer concerns regarding cholesterol contents and lactose intolerance issues, leading to the emergence of commercial non-dairy probiotic beverages [3]. Non-dairy probiotic beverages are commonly fermented from food matrices, such as fruits, cereals or soy [5,6].

Food wastage is undeniably a growing global issue, with up to one-third of all foods produced globally discarded before consumption [7]. Among the different types of food waste, bread is one of the most wasted items [8,9]. In 2013, about 10 million tons of bread waste were generated worldwide, accounting for 10% of total bread production [10]. To tackle the issue of high bread wastage, various studies have been carried out in different applications. A simple technology for recycling industrial waste bread in a zero-waste manner is by processing them into animal feed [11]. However, this application does not bring high added value to the products and food companies.

In recent years, biovalorization of waste bread has been studied, utilizing bread as a substrate for fermentation processes to produce high value-added products [9]. Examples of waste bread biovalorization through fermentation to generate industrial goods include: (A) Biohydrogen produced through anaerobic fermentation [12], (B) succinic acid produced by *Actinobacillus succinogenes* [13], (C) amylases and proteases produced by *Aspergillus awamori* [8], (D) ethanol produced by *Saccharomyces cerevisiae* [10], and kvass, a low-alcohol drink (≤1.2%, *v*/*v*), which is normally produced by *S. cerevisiae* and recently by combination with lactic acid bacteria (LAB, e.g., *Lactobacillus casei*) [14]. Of note, the aforementioned strategies all leave behind substantial amounts of solid waste as most of the solid bread and cell biomass is not incorporated into the final products, but rather centrifuged or filtered out. Apart from these explored applications, there is also the potential of fermenting bread to produce functional probiotic beverages, which are high value-added consumer products. Particularly, this is a zero-waste approach that can fully incorporate the bread into the final products. This application also has the potential to yield a diversity of products, with possible variations in many factors, including but not limited to bread types, bread treatments, probiotic strains, and flavor modulations.

*Lacticaseibacillus rhamnosus* GG (LGG) is a well-characterized strain that is recognized as safe for consumption in commercial supplements and food products [15]. Health benefits delivered by LGG include prevention and treatment of diarrhoea and gastrointestinal infections, improvement of immune responses, and prevention of certain allergic symptoms [15]. In addition, previous studies have indicated the potential of LGG in delivering postbiotic and paraprobiotic effects through immunomodulatory, anti-inflammatory, anti-proliferative, and pro-apoptotic activities in the in vitro and animal studies [2]. Although LGG is reported to have the ability to survive the acidic environment of the stomach and bile digestion to colonize the gastrointestinal tract [16], its viability can still be damaged due to a combination of pH reduction, temperature fluctuations, and oxygen toxicity [17]. Viability reduction is undesirable as it can diminish the health benefits and shorten the shelf life of LGG probiotic products. To mitigate the viability-damaging effects of the environment on probiotic *L. rhamnosus* strains, various approaches have been investigated, such as cell microencapsulation and co-culturing with other microorganisms. Studies have shown that the viability of certain strains of *L. rhamnosus* at 30 °C can be enhanced by co-culturing with *Saccharomyces cerevisiae* [18] or *Candida krusei* and *Yarrowia lipolytica* in milk media [18]. In addition, the yeasts may not only contribute to the survival of LAB, but also enrich the flavor property of the end product [5,19].

*S. cerevisiae* CNCM I-3856 is one of a few strains of yeasts known to have probiotic properties. It is also clinically proven to relieve intestinal problems in individuals with irritable bowel syndrome [20]. In vitro and animal studies have demonstrated the strain’s anti-infectious properties against enterotoxigenic *Escherichia coli* H10407 [21], anti-fungal and anti-inflammatory effects against vaginal candidiasis [22], as well as anti-microbial effects against bacterial vaginosis [23,24]. In addition, its probiotic property, *S. cerevisiae* CNCM I-3856 was included for the study of its interactions with LGG, since co-culturing *S. cerevisiae* EC-1118 with *L. rhamnosus* HN001 has been shown to improve the viability of the latter [18]. It was hypothesized that yeasts can enhance probiotic viability by providing enzymatic and non-enzymatic antioxidants (e.g., aromatic amino acids, peptides) that can act against oxygen toxicity, and acting as a source of nutrients, such as amino acids. In addition, yeast parietal polysaccharides may enhance probiotic viability by providing carbon sources for energy production, as well as creating a physical barrier around the bacterial cells to shield them from the adverse environment [25].

Therefore, the aim of this study was to explore the biovalorization of market surplus bread through fermentation of bread slurries with *L. rhamnosus* GG and *S. cerevisiae* CNCM I-3856 to produce probiotics-fermented functional beverages, with the intention of reducing food wastage by upcycling surplus bread to produce high value-added products. The use of *L. rhamnosus* GG and *S. cerevisiae* CNCM I-3856 in the present study is mainly due to their potential probiotic properties as well as their clinical trials [22,23,24]. 

## 2. Materials and Methods

### 2.1. Microorganisms

The microorganisms used were *Lacticaseibacillus rhamnosus* GG ATCC 53103 (LGG), and *Saccharomyces cerevisiae* CNCM I-3856 (both from Gnosis by Lesaffre, Marcq-en-Baroeul, France). The I-3856 strain of *Saccharomyces cerevisiae* is a proprietary, well-characterized strain of Lesaffre, registered in the French National Collection of Cultures of Microorganisms (CNCM). Both probiotic cultures were provided by Lesaffre Asia Pacific Pte Ltd. in the freeze-dried form and active dried form for LGG and *S. cerevisiae*, respectively. LGG was propagated by inoculating the freeze-dried culture into de Man, Rogosa and Sharpe (MRS) broth (Oxoid Ltd., Hampshire, UK) and incubating at 37 °C for 48 h. *S. cerevisiae* CNCM I-3856 was propagated by inoculating the active-dried culture into yeast-malt (YM) broth comprising of 10 g/L dextrose (Sigma-Aldrich, St. Louis, MO, USA), 3 g/L yeast extract, 3 g/L malt extract, and 5 g/L bacteriological peptone (all from Oxoid Ltd.) and incubating at 30 °C for 48 h. Propagated cultures of LGG and *S. cerevisiae* CNCM I-3856 were aliquoted into cryovials with 15% (*v*/*v*) glycerol (Merck, Darmstadt, Germany), and stored at −80 °C prior to use.

### 2.2. Preparation of Bread Slurries as Fermentation Media

The bread variants explored were enriched white bread (EWB), fine grain wholemeal bread (FGWB), and high calcium milk bread (HCMB) (all from Gardenia, Singapore) in sliced loaf form and were purchased from a local supermarket. Ingredients of bread variants and their nutritional information (which can also be significantly affected by the age of the breads) can be found in Tables S1 and S2. With regards to bread slurry preparation, bread slices from the bread loaf (excluding the two slices from two ends of the loaf) were cut into small dices. The moisture content of the bread dices was measured with an MOC-120H moisture analyzer (Shimadzu, Kyoto, Japan). Based on the measured moisture content, the bread dices were topped with Ice Mountain mineral water (Fraser and Neave Ltd., Singapore) to obtain the specified total solids content. The mixture was homogenized using a Silverson L4RT mixer with an Emulsor Screens workhead (Silverson Machines Ltd., Buckinghamshire, UK) at 7000 rpm for 15 min. Then, the slurry was sterilized at 121 °C for 15 min (optimized condition), and cooled down to room temperature. The prepared slurry was refrigerated at 4 °C for use in sub-culturing the probiotics or fermentation within 3 days.

### 2.3. Microbial Enumeration

Samples were serially diluted with maximum recovery diluent comprising of 1 g/L bacteriological peptone (Oxoid Ltd.) and 8.5 g/L NaCl (Goodrich Chemical Enterprises, Singapore), followed by plating the appropriate dilutions on selective agar. Viable LGG cell counts were determined via the pour plate method using MRS agar supplemented with 5 g/L of Natamax (Danisco A/S, Copenhagen, Denmark) as an anti-fungal agent. The plates were incubated at 37 °C for 48 h prior to counting. Viable *S. cerevisiae* CNCM I-3856 cell counts were determined via the spread plate method using potato dextrose agar (PDA, Oxoid Ltd.) supplemented with 0.1 g/L of chloramphenicol (Sigma-Aldrich, St. Louis, MO, USA) as an anti-bacterial agent (effectiveness confirmed in preliminary trials). The plates were incubated at 30 °C for 48 h prior to counting.

### 2.4. Fermentation of Bread Slurries with Probiotics

Bread slurries of 1.25, 2.5, and 5.0% (*w*/*w*, dry weight) total solids contents made from EWB were prepared as described above. These slurries demonstrated flowable consistency that was deemed suitable for beverage applications. To obtain probiotic starter cultures, LGG (37 °C, 24 h) and *S. cerevisiae* CNCM I-3856 (30 °C, 24 h) frozen stock cultures were sub-cultured twice in a bread slurry (5% inoculation, *v*/*w*) to allow for stabilization of cell counts.

With regards to mono-culture fermentations, bread slurries in Schott bottles were inoculated with 1% (*v*/*w*) of LGG or *S. cerevisiae* CNCM I-3856 starter culture. With regards to the co-culture, both LGG and yeast starter cultures were inoculated at 1% (*v*/*w*). The inoculated samples were mixed thoroughly and aliquoted into 50-mL centrifuge tubes (40 mL in each tube) for incubation at 37 °C. At 0, 16, 20, 24, 48, and 72 h, samples were subjected to pH measurement (Five Easy Plus pH meter, Mettler Toledo, Giessen, Germany) and microbial enumeration.

### 2.5. Fermentation of Bread Slurries Made from Different Bread Variants

The bread slurries were made from EWB, FGWB, and HCMB of 5.0% (*w*/*w*) total solids content. After inoculation and incubation, samples were subjected to pH measurement and microbial enumeration at 0 and 16 h of incubation.

### 2.6. Fermentation of Bread Slurries Supplemented with Sweeteners and Stabiliser

The bread slurry was made from EWB of 5.0% (*w*/*w*) total solids bread content. Prior to sterilization, the blended bread slurry was added with a zero-calorie sweetener mix (erythritol—99.5%, steviol glycosides, vanilla extract) from Taikoo Sugar Refinery (Hong Kong) at 3% (*w*/*w*) and with Kelcogel^®^ Gellan Gum stabilizer (CP Kelco, Atlanta, GA, USA) at 0.001% (*w*/*w*) (based on preliminary trials and suppliers’ recommendation). The additives were added during mixing with a Silverson L4RT mixer at 3000 rpm for 1 min followed by further blending at 5000 rpm for 10 min. After sterilization at 121 °C for 15 min, slight shaking was applied to the slurry upon cooling down to ambient temperature for dispersion of the stabilizer. 

Fermentation was carried out following the established protocol. The bread slurry used for sub-culturing was made from EWB of 5.0% (*w*/*w*) total solids content, without additives. After inoculation and incubation, samples were subjected to pH measurement and microbial enumeration at 0, 16, and 24 h.

### 2.7. Shelf Life Monitoring

Shelf life monitoring was carried out for samples prepared as described above. After inoculation and incubation at 37 °C for 16 h, fermented samples were transferred to 50-mL centrifuge tubes (40 mL in each tube) for storage at 5 and 30 °C. Shelf life samples were monitored with weekly pH measurement and microbial enumeration. Unfermented, fermented, and end of shelf life fermented samples were further analyzed for sugars, organic acids, free amino acids, volatile compounds, and ethanol contents.

### 2.8. Non-Volatile Compounds Analysis

Sugars in samples were extracted by diluting 1 g of sample with 2 mL of acetonitrile (Tedia, Fairfield, OH, USA) and vortexing for 1 min. The mixtures were centrifuged at 20,000× *g* for 20 min at 4 °C and filtered through a 0.2-μm Minisart RC 15 syringe filter (Sartorius, Goettingen, Germany) to obtain the sugar extracts. Liquid chromatography analysis of sugars (glucose and fructose) was performed with a Shimadzu Prominence UFLC system, as reported previously [5]. 

With regards to organic acids, samples were prepared by diluting 1 g of sample with 3 mL of 0.1% (*v*/*v*) H_2_SO_4_. The mixtures were vortexed, centrifuged, and filtered to obtain the organic acids extracts. Chromatographic separation was performed at 40 °C using a Supelcogel C-610H column (Supelco, Bellefonte, PA, USA) and 0.1% (*v*/*v*) H_2_SO_4_ mobile phase with a flow rate of 0.4 mL/min. Organic acids were detected at 210 nm with an SPD-M20A photodiode array detector. 

Samples for free amino acid (FAAs) analysis were prepared by diluting 1 g of sample with 250 μL of 10% salicylic acid (Sigma-Aldrich) and vortexing for 1 min. The mixtures were centrifuged at 20,000× *g* for 5 min at 4 °C and filtered to obtain the FAAs extracts. Separation of FAAs was performed using an Aracus Amino Acid Analyzer (membraPure GmbH, Berlin, Germany) [26]. Separated FAAs were derivatized post-column with ninhydrin and detected with LED photometers at 570 and 440 nm. Amino acid physiological standards (membraPure GmbH) were used for identification and quantification of FAAs.

### 2.9. Volatile Organic Compounds Analysis

Analysis of volatile organic compounds (VOCs) in samples was carried out with a headspace-solid-phase micro-extraction-gas chromatography-mass spectrometer/flame ionization detector (HS-SPME-GC-MS/FID) system. The sample (5 g) was added with 2 g of NaCl to a 20-mL glass headspace vial sealed with a polytetrafluoroethylene (PTFE) septum. The sample vial was incubated at 60 °C for 20 min, and VOCs in the sample’s headspace were extracted with an 85-μm carboxen/polydimethylsiloxane (CAR/PDMS) SPME fiber (Supelco, Sigma-Aldrich, Barcelona, Spain) at 60 °C for 30 min with 250 rpm agitation using a Combi Pal autosampler (CTC Analytics, Zwingen, Switzerland). The SPME fiber was thermally desorbed at 250 °C for 3 min in the injection port of an Agilent 7890A gas chromatograph coupled to an Agilent 5975C triple-axis MS and FID. VOCs were separated with a DB-FFAP capillary column (60 m length, 0.25 mm i.d., 0.25 µm film thickness, Agilent) and identified by matching their mass spectra with the NIST 08 and Wiley 275 databases, as well as confirmed with their linear retention index (LRI) value.

### 2.10. Ethanol Contents Analysis

Fermented samples (40 mL) were centrifuged at 20,000× *g* for 10 min at 4 °C, and the ethanol contents of the supernatants were measured using the Alcolyzer ME alcohol measuring module coupled with a DMA™ 4500 M density meter (Anton-Parr GmbH, Baden-Wurttemberg, Germany).

### 2.11. Statistical Analysis

All of the data were expressed as the mean ± standard deviation obtained from three independent experiments (*n* = 3). One-way analysis of variance (ANOVA) and Duncan’s multiple range test with SPSS^®^ 20.0 (SPSS Inc., Chicago, IL, USA) were used for significant differences analysis at *p* < 0.05.

## 3. Results and Discussion

### 3.1. Fermentation of Bread Slurries with Probiotics 

**Figure 1:**
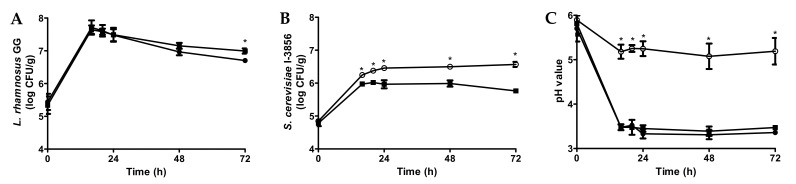
Changes in viable *L. rhamnosus* GG (**A**) and *S. cerevisiae* CNCM I-3856 (**B**) cell counts and changes in pH (**C**) during 37 °C incubation in bread slurries (2.5% total solids) inoculated with *L. rhamnosus* GG only (●), *S. cerevisiae* CNCM I-3856 only (○), and *L. rhamnosus* GG + *S. cerevisiae* CNCM I-3856 (■) propagated in bread slurry. * Indicates significant differences (*p* < 0.05) within the same time point.

The growth of probiotics in 2.5% bread slurry is shown in Figure 1. LGG grew from 5.4 to 7.7 log CFU/mL within 16 h at 37 °C for both mono-culture and co-culture samples (Figure 1A). The cell counts remained relatively stable from 16 to 24 h, followed by a significant decline (*p* < 0.05) from 24 to 72 h for both cultures. LGG co-cultured with *S. cerevisiae* CNCM I-3856 showed a significantly higher viability compared to the mono-culture at 72 h (Figure 1A). With regards to the probiotic yeast, bread slurries were inoculated with 4.8 log CFU/mL of *S. cerevisiae* CNCM I-3856 (Figure 1B). During incubation at 37 °C, viable yeast cell counts peaked at 6.5 log CFU/mL (24 h) in mono-culture and at 6.0 log CFU/mL (20 h) when co-cultured with LGG. It was evident that *S. cerevisiae* CNCM I-3856 cell counts in mono-culture were significantly higher compared to the co-cultured samples during the whole period of fermentation.

The maximum LGG cell count (7.7 log CFU/mL) was slightly higher than the general guideline for typical probiotic beverages (7 log CFU/mL). However, the maximum *S. cerevisiae* CNCM I-3856 cell count in mono-culture (6.5 log CFU/mL) was lower and unable to deliver the dosage used in clinical studies that demonstrated probiotic effects (9.0 log CFU/serving) in a realistic beverage serving size [20]. The low cell counts might be attributed to the fact that bread slurry is not the ideal substrate for yeast growth due to its low sugar content (Table 1). In addition, the incubation temperature (37 °C) used was optimized for LGG growth, while the typical optimal temperature range for growth of *S. cerevisiae* is about 30 °C. The lower peak cell count (6.0 log CFU/mL) of *S. cerevisiae* CNCM I-3856 in samples co-cultured with LGG can be explained by competition for nutrients and quorum sensing. Furthermore, LGG can produce metabolites, such as bacteriocins and acids, the acids are detrimental to yeast viability. In particular, the production of reactive oxygen species, such as hydrogen peroxide can introduce oxygen toxicity, while the production of organic acids, such as lactic and acetic acids can lead to the reduction of pH to levels toxic to the yeast [17]. The contribution of LGG metabolic activities to the acidic environment was evident in the co-culture samples with considerably lower pH (3.4) compared to *S. cerevisiae* CNCM I-3856 mono-culture samples (pH 5.8) (Figure 1C). In addition, co-culturing the yeasts with LAB may enrich the flavor properties of the end products compared to its mono-culture fermentation [5,14,19].

### 3.2. Fermentation of Bread Slurries of Different Bread Concentrations

**Figure 2:**
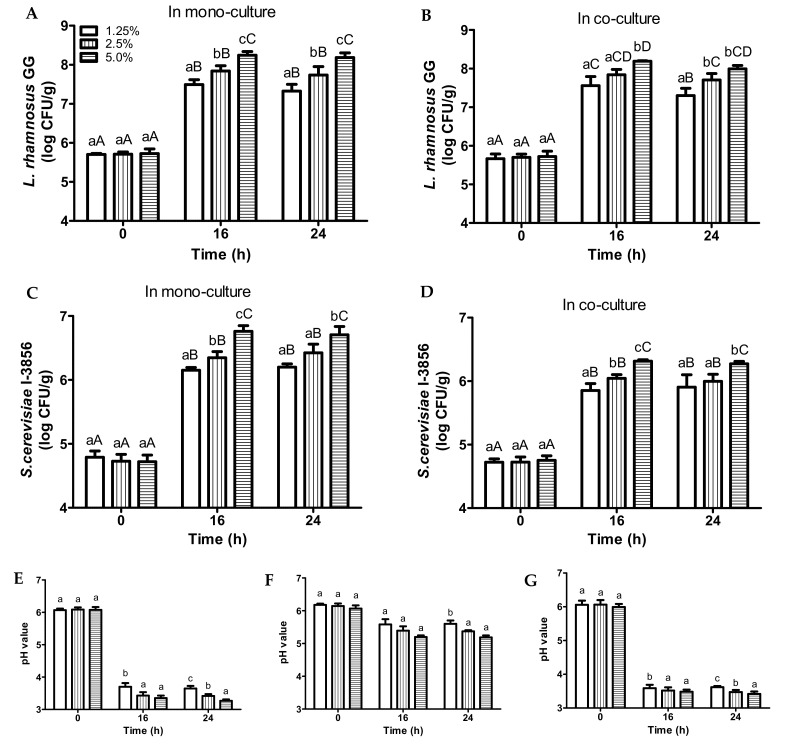
Changes in viable *L. rhamnosus* GG and *S. cerevisiae* CNCM I-3856 cell counts during 37 °C incubation in bread slurries inoculated with *L. rhamnosus* GG only (**A**), *L. rhamnosus* GG + *S. cerevisiae* CNCM I-3856 (**B**), *S. cerevisiae* CNCM I-3856 only (**C**), and *L. rhamnosus* GG + *S. cerevisiae* CNCM I-3856 (**D**). Changes in pH during 37 °C incubation for bread slurries inoculated with *L. rhamnosus* GG only (**E**), *S. cerevisiae* CNCM I-3856 only (**F**), and *L. rhamnosus* GG + *S. cerevisiae* CNCM I-3856 (**G**). Mean values at the same time point with different lower case letters are significantly different (*p* < 0.05). Mean values at the different time points with different upper case letters are significantly different (*p* < 0.05).

With regards to LGG, all of the EWB bread slurries (1.25%, 2.5%, 5.0%, *w*/*w*) were inoculated with 5.7 log CFU/mL. After 16 h, LGG cell counts were at their maximum with significant differences between the different bread concentrations (Figure 2A,B). The extent of LGG growth significantly increased with the increasing bread contents. In addition, maximum LGG cell counts (16 h) in both mono-culture and co-culture samples at each initial solid bread concentration were almost the same with 1.25% (7.5, 7.6 log CFU/mL), 2.5% (7.8, 7.8 log CFU/mL), and 5.0% (8.2, 8.2 log CFU/mL), respectively (Figure 2A,B).

Similar trends were observed for *S. cerevisiae* CNCM I-3856 (Figure 2C,D), all of the samples were inoculated with 4.7 log CFU/mL. With regards to mono-culture, *S. cerevisiae* CNCM I-3856 cell counts (16 h) in samples of 1.25%, 2.5%, 5.0% initial solid bread contents were 6.2, 6.4, and 6.8 log CFU/mL, respectively (Figure 2C). With regards to co-culture, *S. cerevisiae* CNCM I-3856 cell counts (16 h) in samples were 5.9 (1.25%), 6.1 (2.5%), and 6.3 (5.0%) log CFU/mL, respectively (Figure 2D). It was notable that higher viable *S. cerevisiae* CNCM I-3856 cell counts were obtained in mono-culture samples compared to co-culture samples.

With regards to pH values (Figure 2E–G), pH changes in samples of different initial bread contents were comparable. In some instances, the extent of pH drops in samples slightly increased (*p* < 0.05) with increasing initial bread contents.

It was observed that higher bread concentrations resulted in better growth of the probiotics and higher peak cell counts, as expected due to the higher amount of nutrients supplied to the probiotics. Among the investigated bread concentrations, a bread slurry of 5.0% initial total bread solids yielded the highest viable cell counts for both LGG and *S. cerevisiae* CNCM I-3856, this concentration was used in subsequent fermentations. Bread slurries with concentrations higher than 5.0% were not explored as the texture of the slurries would be too thick and viscous for beverage applications (Table S3).

### 3.3. Fermentation of Bread Slurries Made from Different Bread Variants

**Figure 3:**
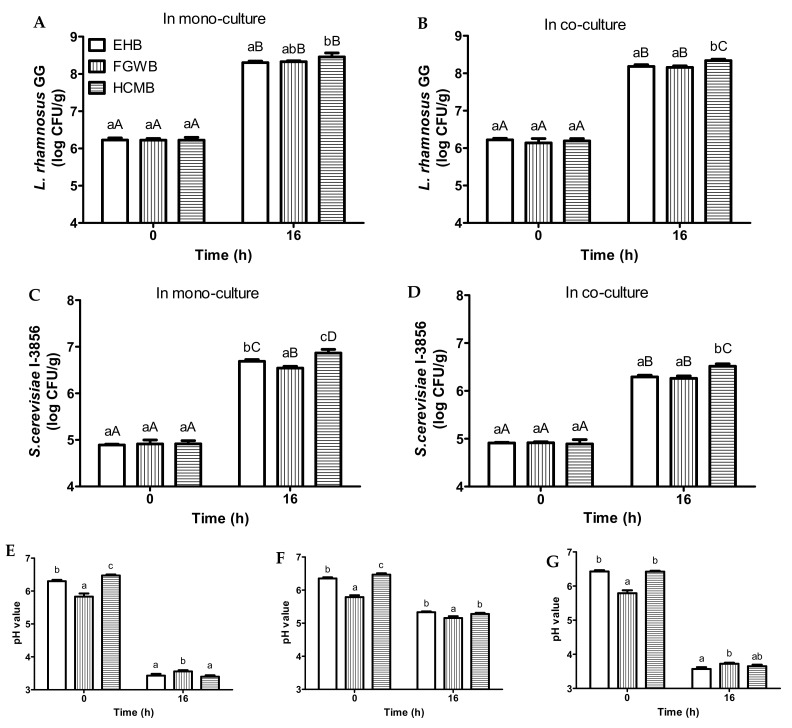
Changes in viable *L. rhamnosus* GG cell counts in monoculture (**A**) and co-culture (**B**), *S. cerevisiae* CNCM I-3856 cell counts in monoculture (**C**) and co-culture (**D**). Changes in pH during 37 °C incubation for bread slurries inoculated with *L. rhamnosus* GG only (**E**), *S. cerevisiae* CNCM I-3856 only (**F**), and *L. rhamnosus* GG + *S. cerevisiae* CNCM I-3856 (**G**) in samples made from 5.0% total solids of enriched white bread (EHB), fine grain wholemeal bread (FGWB), and high calcium milk bread (HCMB). Mean values at the same time point with different lower case letters are significantly different (*p* < 0.05). Mean values at the different time points with different upper case letters are significantly different (*p* < 0.05).

With regards to LGG fermented samples, all of the bread slurries were inoculated with 6.2 log CFU/mL of LGG and incubated at 37 °C for 16 h (Figure 3A,B). With regards to mono-culture samples, LGG cell counts increased to 8.3 (EWB), 8.3 (FGWB), and 8.5 (HCMB) log CFU/mL, respectively (Figure 3A). With regards to co-culture samples, LGG cell counts increased to 8.2 (EWB), 8.2 (FGWB), and 8.3 (HCMB) log CFU/mL, respectively (Figure 3B).

With regards to yeast fermented samples, all of the bread slurries were inoculated with 4.9 log CFU/mL of *S. cerevisiae* CNCM I-3856 and incubated at 37 °C for 16 h (Figure 3C,D). With regards to mono-culture samples, yeast cell counts increased to 6.7, 6.5, and 6.9 log CFU/mL for samples made from EWB, FGWB, and HCMB, respectively (Figure 3C). With regards to co-culture samples, yeast cell counts increased to 6.3 (EWB), 6.3 (FGWB), and 6.5 (HCMB) log CFU/mL, respectively (Figure 3D). With regards to pH values, slight variations were observed in samples made from different bread variants (Figure 3E–G).

Our results indicated that the production of probiotic bread beverages was feasible on various types of bread. It should be noted that the variants of the investigated bread were all wheat bread. It was expected that bread loafs purchased from other brands would possess similar properties, and hence would also be able to support microbial growth. It was observed that probiotic growth was mostly comparable in bread slurries made from FGWB and EWB. On the other hand, probiotic growth in bread slurries made from HCMB was slightly higher as compared to the other two bread types (Figure 3). This can be attributed to HCMB containing inulin (Table S1), which some strains of *S. cerevisiae* might be able to utilize as a nutrient source due to the presence of the enzyme invertase SUC2 [27]. In addition, even though inulin is not metabolizable by LGG, it can be degraded under the acidic condition in LGG fermented bread slurries to release fructose, which can be used as an additional energy source for the probiotics [28]. Furthermore, the higher calcium content in HCMB (Table S2) might have a buffer capacity, which would have contributed to the increased protection from microbial damage by maintenance of membrane permeability barrier of the probiotics, through association of the calcium ions on the surface of the microbial cells [29].

### 3.4. Fermentation of Bread Slurries Supplemented with Additives

To enhance the organoleptic properties of the fermented probiotic bread beverages, 3% zero-calorie sweetener and 0.001% Kelcogel^®^ Gellan Gum stabilizer (*w*/*w*) were added into 5.0% EWB slurries before fermentation. The fermented samples were found to exhibit added sweetness (qualitative sensory assessment) and delayed phase separation (Table S4). The added gellan gum was hydrated upon the heat treatment that resulted in swelling of the gellan gum molecules. This led to an increase in viscosity of the sample, and a formation of a weak gel structure. The increase in viscosity and the formation of gel structure contributed to the delayed phase separation by delaying particle movements due to Brownian motion or gravity pull. This helped with the holding of particles in their place in the sample suspension [30].

**Figure 4:**
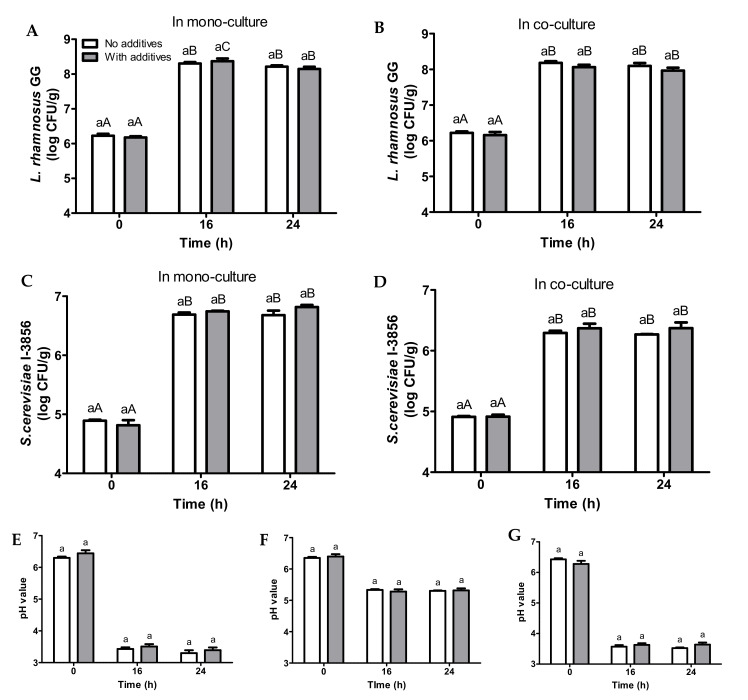
Changes in viable *L. rhamnosus* GG cell counts in mono-culture (**A**) and co-culture (**B**), *S. cerevisiae* CNCM I-3856 cell counts in monoculture (**C**) and co-culture (**D**). Changes in pH during 37 °C incubation for bread slurries inoculated with *L. rhamnosus* GG only (**E**), *S. cerevisiae* CNCM I-3856 only (**F**), and *L. rhamnosus* GG + *S. cerevisiae* CNCM I-3856 (**G**) during 37 °C incubation for bread slurries made from 5.0% total solids of EWB without additives or with additives (3% sweetener + 0.001% stabilizer). Mean values at the same time point with different lower case letters are significantly different (*p* < 0.05). Mean values at the different time point with different upper case letters are significantly different (*p* < 0.05).

No differences in LGG and *S. cerevisiae* CNCM I-3856 cell counts were observed between the samples with and without additives (Figure 4). Peak cell counts for all of the samples were observed after 16 h of incubation at 37 °C. Regarding samples supplemented with additives, peak LGG cell counts were 8.4 log CFU/mL in mono-culture (Figure 4A) and 8.1 log CFU/mL for co-culture (Figure 4B) samples. With additives, peak *S. cerevisiae* CNCM I-3856 cell counts were 6.7 log CFU/mL in mono-culture (Figure 4C) and 6.4 log CFU/mL for co-culture (Figure 4D) samples. With regards to pH (Figure 4E–G), no differences were observed between the samples with and without additives. 

Overall, the supplementation of additives did not affect probiotic growth in bread slurries, as all of the additives were neither fermentable by LGG nor *S. cerevisiae* CNCM I-3856. It could be concluded that the increase in viscosity, the introduction of a gel structure, as well as changes in particle distribution in the bread slurry due to delayed particle movements did not affect microbial access to nutrients and exposure to inhibitory metabolites.

### 3.5. Shelf Life Monitoring of Fermented Bread Beverages

**Figure 5:**
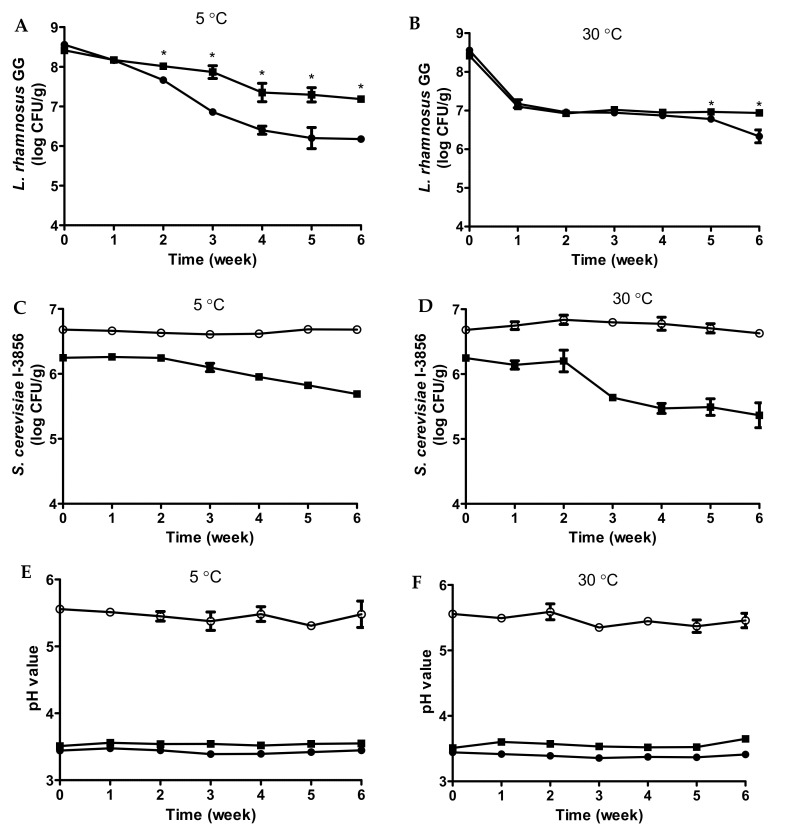
Changes in viable *L. rhamnosus* GG cell counts during storage at 5 (**A**) and 30 °C (**B**). Changes in viable *S. cerevisiae* CNCM I-3856 cell counts during storage at 5 (**C**) and 30 °C (**D**). Changes in pH during storage at 5 (**E**) and 30 °C (**F**) for fermented bread beverages inoculated with *L. rhamnosus* GG monoculture (●), *S. cerevisiae* CNCM I-3856 (○), and co-culture (■) followed by 37 °C incubation for 16 h. * Indicates significant differences (*p* < 0.05) at the same time point.

At the beginning of shelf life (after fermenting at 37 °C for 16 h), viable LGG cell counts were 8.6 log CFU/mL in mono-culture samples and 8.4 log CFU/mL in co-culture samples (Figure 5A,B). At 5 °C storage, a significant reduction in LGG cell counts was noted after 1 week for both mono-culture and co-culture samples (Figure 5A). Subsequently, LGG cell counts declined sharply in mono-culture samples compared to co-culture. Significant differences in LGG cell counts between mono-culture and co-culture samples started to be revealed at week 2, with co-culture samples having 0.4 log CFU/mL higher than the mono-culture samples. At the end of the monitoring period (week 6), co-culture samples had 7.2 log CFU/mL of LGG, which was 1.0 log CFU/mL higher than the mono-culture samples (6.2 log CFU/mL). At 30 °C storage, a significant and sharp decline of LGG cell counts was recorded after 1 week of storage for both mono-culture and co-culture samples (Figure 5B). Subsequently, LGG cell counts remained relatively stable for co-culture samples and gradually declined for mono-culture samples. Significant differences in LGG cell counts between mono-culture and co-culture samples started to be displayed at week 5. At week 6, co-culture samples had 6.9 log CFU/mL of LGG, which was 0.6 log CFU/mL higher than mono-culture samples (6.3 log CFU/mL).

A significant reduction in LGG cell counts was found in samples throughout shelf life, which can be mainly attributed to acid toxicity. During fermentation and storage, lactic acid was produced in the cytoplasm of LGG cells as an end-product of glycolysis, which could be transported out of the cells via facilitated diffusion with lactate-proton symporters, causing a reduction in the medium pH [31]. This led to increased protonation, thereby increasing the proportion of organic acids in the non-dissociated form. Non-dissociated acids could diffuse passively across the cell membrane and dissociate in the more alkaline cytoplasm, lowering the intracellular pH [32]. The lowering of intracellular pH and accumulation of anions disrupted the metabolic processes that are crucial for the survival of the probiotic, leading to a reduction in LGG viability [33].

The viability of LGG in co-culture samples was better than mono-culture samples (Figure 5A,B). Regarding co-culture samples, LGG cell counts were at around 7 log CFU/mL after 6 weeks of storage for both refrigerated and elevated temperatures, qualifying the general guidelines for probiotic beverages. Better viability of LAB was similarly reported in co-culturing *S. cerevisiae* EC-1118 with *L. rhamnosus* in an acidic environment [18]. With regards to mono-culture samples, it was interesting to note that LGG cell counts at 30 °C had a sharp reduction at week 1, but then remained relatively stable (Figure 5B). On the other hand, LGG cell counts at 5 °C were observed with gradual reduction throughout shelf life. Starting from week 3, mono-culture samples had higher LGG cell counts at 30 °C compared to 5 °C, which was unexpected. This observation is hypothesized to be explained by different sugar metabolisms by LGG at different storage temperatures.

With regards to yeast cell counts, at the beginning of shelf life (after fermenting at 37 °C for 16 h), viable *S. cerevisiae* CNCM I-3856 cell counts were 6.7 log CFU/mL in mono-culture samples (Figure 5C) and 6.3 log CFU/mL in co-culture samples (Figure 5D). At 5 °C storage, yeast cell counts remained relatively stable for mono-culture samples (Figure 5C). However, a gradual reduction was observed in co-culture samples starting from week 3. At the end of the monitoring period (week 6), co-culture samples had only 5.7 log CFU/mL of *S. cerevisiae* CNCM I-3856, which was 1.0 log CFU/mL lower than mono-culture samples (6.7 log CFU/mL). At 30 °C storage, yeast cell counts remained relatively stable for mono-culture samples (Figure 5D), which is similar to the 5 °C storage. On the contrary, a sharp reduction in yeast cell counts was observed in co-culture samples at week 3, followed by a gradual reduction. At week 6, co-culture samples had only 5.4 log CFU/mL of *S. cerevisiae* CNCM I-3856, which was 1.2 log CFU/mL lower than mono-culture samples (6.6 log CFU/mL).

The stability of yeast cell counts in mono-culture can be attributed to the absence of acid toxicity. In addition, the yeast cells were not challenged with ethanol toxicity as the ethanol contents in fermented samples were all below 0.5% (*v*/*v*) (Table S5). On the other hand, a reduction in yeast cell counts in co-culture samples throughout shelf life was likely due to the competition for resources with LGG and the introduction of toxic metabolites by LGG. Nevertheless, although at the end of 6 weeks, *S. cerevisiae* CNCM I-3856 cell counts in co-culture samples were lower, the addition of yeasts was beneficial in sustaining LGG viability and extending the shelf life of the probiotic beverages. In addition, there are possibilities that *S. cerevisiae* CNCM I-3856 even at low viable cell counts can exhibit beneficial postbiotic and paraprobiotic effects, as well as probiotic effects in synergy with LGG [34].

The pH values of shelf life samples remained relatively stable throughout storage at around 3.4 for LGG mono-culture samples (Figure 5E), 5.5 for *S. cerevisiae* CNCM I-3856 mono-culture samples (Figure 5F), and 3.6 for co-culture samples (Figure 5G).

As shown in Table 1, unfermented bread slurries contained fructose and glucose. After fermentation (37 °C, 16 h), glucose was completely utilized in yeast-fermented samples. Fructose was partially utilized during fermentation and completely consumed at the end of shelf life. Regarding LGG mono-culture fermented samples, glucose was exhausted, while fructose was partially utilized. No differences in sugar contents were observed at week 6 compared to week 0 for 5 °C storage. However, complete utilization of fructose was observed at week 6 for 30 °C storage. It might be possible that under temperature stress, LGG could further metabolize sugars. This ability might have been the reason for better LGG viability at 30 °C storage compared to 5 °C storage for mono-culture samples.

With regards to organic acids, oxalic, malic, acetic, and propionic acids were identified in unfermented bread slurry (Table 1). Throughout fermentation and shelf life, malic acid was utilized by both LGG and yeast. Regarding LGG, malic acid might have been decarboxylated to lactic acid by malolactic enzyme [35]. Regarding *S. cerevisiae* CNCM I-3856, malic acid was likely decarboxylated by malic enzyme to pyruvate or by malate dehydrogenase to oxaloacetate LGG produced lactic acid mainly through the Embden-Meyerhof-Parnas pathway [30], contributing to the low pH of LGG fermented samples. During storage, there were slight increases in lactic acid for mono-culture samples and in acetic acid for both mono-culture and co-culture samples (Table 1). In addition, an increase in acetic acid was also observed in yeast mono-culture samples, possibly as a by-product of alcoholic fermentation under oxidative stress, where acetaldehyde was oxidized to acetic acid by aldehyde dehydrogenase. No post-acidification was observed during shelf life monitoring of yeast fermented samples, even though there were slight increases of acetic acid.

With regards to FAAs, an increase in overall FAAs contents was observed for LGG fermented samples (Table 1), as lactic acid bacteria can carry out proteolysis to produce amino acids, which are needed as their nutrient source [36]. In addition, there were increases in γ-aminobutyric acid (GABA) contents during shelf life of LGG mono-culture (5.84 μg/mL, week 6) and co-culture samples (12.03 μg/mL, week 6), as LGG has glutamate decarboxylase that allows for the production of GABA via the GABA shunt. The production of GABA could be an acid tolerance mechanism, where glutamate is decarboxylated and captures a proton in the environment [37]. The increases in GABA in LGG fermented samples (both mono-culture and co-culture samples) may present potential nutritional benefits due to its well-documented therapeutic effects. In addition, increases in ammonia contents were observed in LGG mono-culture and co-culture samples at 30 °C storage, which were likely produced by LGG in response to acidic stress, as ammonia is slightly basic. It has been reported that ammonia can be produced from the arginine deamination pathway through the conversion of arginine into citrulline [38].

As opposed to LGG fermented samples, a reduction in FAAs contents was observed in yeast mono-culture fermented samples as yeast utilizes amino acids as nitrogen sources for biomass production [39]. The FAAs contents slightly increased in samples stored at 30 °C, which can be due to the release of FAAs from yeast autolysis under stress conditions, de novo biosynthesis of amino acids [40] or release of amino acids from proteins by yeast proteases and peptidases.

Identified VOCs are summarized in Table S6. Acetic acid and propionic acid increased after fermentation and during shelf life, which corresponded with HPLC analysis (Table 1). The production of butyric acid by LGG was observed, which was not detected in HPLC analysis, likely due to the fact that concentrations of butyric acid in samples were below the limit of detection. 

With regards to alcohols, endogenous ethanol was detected in unfermented bread slurry, likely as residual ethanol from bread making. Significant ethanol production was observed in yeast fermented samples. However, all of the fermented samples had ethanol contents lower than 0.5% *v*/*v* (Table S5) and were considered non-alcoholic. The samples were also characterized by the presence of isobutyl alcohol, which was produced from valine via the Ehrlich pathway [5,26]. Yeast mono-culture samples produced 2-phenethyl alcohol, which has a floral, rose-like flavor. On the other hand, LGG fermented samples produced active amyl alcohol and 2-ethyl-1-hexanol, the latter could impart a slightly floral flavor to the products [41].

With regards to ketones and aldehydes, diacetyl was only detected in LGG mono-culture samples. Diacetyl together with 1-heptanone, 2-octanone, and acetoin in all of the samples provided a green, fatty/creamy, buttery flavor. In addition, LGG fermented samples contained furfural, which had an almond and bread flavor [41]. Yeast mono-culture was detected with the production of butyrolactone, which had a cheesy/creamy aroma. Finally, all of the samples were detected with esters, such as ethyl octanoate, which may impart a fruity flavor [41].

## 4. Conclusions

Bread slurries were shown as a suitable substrate for the production of a fermented beverage with mono-culture and co-culture of LGG and *S. cerevisiae* CNCM I-3856. The peak cell counts of LGG were above 8 log CFU/mL for both mono-culture and co-culture samples. However, *S. cerevisiae* CNCM I-3856 was below 7 log CFU/mL in all of the treatments. Peak cell counts for *S. cerevisiae* CNCM I-3856 in co-culture were lower than in mono-culture samples, likely due to the high acidity introduced by LGG. During shelf life monitoring, the viability of LGG was better in co-culture than mono-culture, demonstrating the protective effects on LGG provided by *S. cerevisiae* CNCM I-3856. At both refrigerated and elevated storage temperatures, LGG cell counts in co-culture samples were maintained at around 7 log CFU/mL, but lower than 7 log CFU/mL in mono-culture samples. With regards to *S. cerevisiae* CNCM I-3856, cell counts remained stable throughout shelf life for mono-culture samples, while reductions were observed in co-culture samples. Even though viable *S. cerevisiae* CNCM I-3856 cell counts obtained in co-culture samples were not high, the co-culturing of *S. cerevisiae* CNCM I-3856 with LGG was valuable as it aided in improving LGG viability, and thus helped extend the shelf life of the potential probiotic bread beverages. In addition, the co-culture of *L. rhamnosus* GG and *S. cerevisiae* samples that were stored at 30 °C contained the highest levels of several metabolites, including γ-ABA, leucine, valine, glycine, etc., as well as volatile compounds (e.g., 2-phenethyl alcohol, hexanal).

## Figures and Tables

**Table 1:** Contents of identified sugars, organic acids, and free amino acids in unfermented and fermented bread slurries at the beginning and end of shelf life monitoring.

Compounds	Unfermented Bread Slurry	*L. rhamnosus* GG			*S. cerevisiae* CNCM I-3856			*L. rhamnosus* GG + *S. cerevisiae* CNCM I-3856		
		Week 0	Week 6 (5 °C)	Week 6 (30 °C)	Week 0	Week 6 (5 °C)	Week 6 (30 °C)	Week 0	Week 6 (5 °C)	Week 6 (30 °C)
Sugars (g/L)										
Fructose	3.21 ± 0.11 b	0.85 ± 0.21 a	0.61 ± 0.32 a	ND	0.62 ± 0.34 a	ND	ND	0.48 ± 0.25 a	ND	ND
Glucose	2.46 ± 0.13	ND	ND	ND	ND	ND	ND	ND	ND	ND
Organic acids (g/L)										
Oxalic acid	0.01 ± 0.00 a	0.01 ± 0.00 a	0.01 ± 0.00 a	0.01 ± 0.00 a	0.01 ± 0.00 a	0.01 ± 0.00 a	0.01 ± 0.00 a	0.01 ± 0.00 a	0.01 ± 0.00 a	0.01 ± 0.00 a
Malic acid	0.19 ± 0.03	ND	ND	ND	ND	ND	ND	ND	ND	ND
Lactic acid	ND	2.98 ± 0.20 b	3.17 ± 0.23 bc	3.33 ± 0.13 c	ND	ND	ND	2.50 ± 0.15 a	2.42 ± 0.16 a	2.32 ± 0.41 a
Acetic acid	0.11 ± 0.02 a	0.17 ± 0.02 b	0.15 ± 0.02 b	0.45 ± 0.02 d	0.16 ± 0.05 b	0.15 ± 0.03 b	0.30 ± 0.00 c	0.15 ± 0.03 b	0.15 ± 0.02 b	0.30 ± 0.05 c
Propionic acid	0.18 ± 0.02 a	0.19 ± 0.02 a	0.17 ± 0.03 a	0.19 ± 0.00 a	0.18 ± 0.02 a	0.17 ± 0.01 a	0.18 ± 0.00 a	0.17 ± 0.02 a	0.17 ± 0.01 a	0.18 ± 0.02 a
Free amino acids (μg/mL)										
Ammonia	2.72 ± 0.19 b	2.61 ± 0.52 b	3.26 ± 0.46 b	7.76 ± 0.66 d	1.28 ± 0.10 a	1.10 ± 0.04 a	0.94 ± 0.07 a	1.49 ± 0.09 a	1.59 ± 0.15 a	6.93 ± 1.09 c
Serine	2.00 ± 0.53 a	2.97 ± 0.14 b	3.13 ± 0.16 b	5.63 ± 0.28 c	ND	ND	ND	2.61 ± 0.20 b	2.61 ± 0.39 b	6.67 ± 0.74 d
Glutamic acid	10.13 ± 0.47 a	44.73 ± 6.66 c	43.53 ± 6.49 c	71.34 ± 8.50 c	2.47 ± 0.82 a	1.88 ± 0.35 a	5.37 ± 1.66 a	19.02 ± 2.18 b	19.06 ± 2.46 b	47.96 ± 5.63 c
Glycine	2.04 ± 0.07 b	2.23 ± 0.16 b	2.47 ± 0.20 bc	3.37 ± 0.19 d	ND	ND	1.56 ± 0.74 a	2.08 ± 0.09 b	2.87 ± 0.09 c	4.55 ± 0.28 e
Histidine	ND	ND	ND	1.81 ± 0.20 b	ND	ND	ND	ND	ND	1.60 ± 0.17 a
Arginine	3.51 ± 0.35 b	4.97 ± 0.62 cd	4.88 ± 0.23 cd	5.40 ± 0.39 d	ND	ND	2.04 ± 1.15 a	2.32 ± 0.23 a	2.40 ± 0.29 a	4.36 ± 0.67 bc
Threonine	1.47 ± 0.04 a	ND	ND	ND	ND	ND	ND	ND	ND	3.90 ± 0.61 b
Alanine	12.62 ± 0.33 d	8.79 ± 0.27 c	9.02 ± 0.47 c	13.83 ± 0.70 e	ND	ND	3.74 ± 0.62 a	7.00 ± 0.66 b	8.40 ± 0.77 c	14.00 ± 0.70 e
Proline	1.81 ± 0.14 d	16.70 ± 2.22 c	17.01 ± 2.70 c	25.26 ± 2.85 e	ND	ND	1.78 ± 0.15 a	14.52 ± 1.06 b	13.73 ± 1.35 bc	21.30 ± 1.37 d
Tyrosine	2.29 ± 0.35 a	3.29 ± 0.40 ab	3.95 ± 0.70 b	9.73 ± 0.85 c	ND	ND	2.82 ± 0.35 ab	ND	2.63 ± 0.17 ab	14.79 ± 2.09 c
Valine	1.45 ± 0.61 a	ND	ND	2.72 ± 0.21 b	ND	ND	2.97 ± 0.17 b	ND	ND	7.01 ± 0.52 c
Lysine	2.92 ± 0.27 b	ND	ND	ND	ND	ND	2.38 ± 0.59 a	ND	ND	2.16 ± 0.69 a
Isoleucine	ND	ND	ND	2.52 ± 0.33 a	ND	ND	2.94 ± 0.22 a	ND	ND	6.46 ± 0.97 b
Leucine	2.83 ± 0.16 ab	1.44 ± 0.20 a	1.85 ± 0.25 a	5.84 ± 0.76 c	ND	ND	3.64 ± 0.28 b	ND	1.64 ± 0.18 a	14.76 ± 2.23 c
Tryptophan	5.10 ± 0.18 d	4.38 ± 0.17 c	4.76 ± 0.08 cd	ND	ND	ND	ND	2.32 ± 0.77 a	3.20 ± 0.44 b	ND
γ-ABA	3.51 ± 0.35 cd	2.54 ± 2.21 abc	4.92 ± 0.46 e	5.84 ± 0.74 e	1.67 ± 0.35 ab	1.39 ± 0.21 a	2.70 ± 0.15 abc	3.01 ± 0.45 d	4.81 ± 0.24 e	12.03 ± 0.17 f

Results are expressed as the mean values ± standard deviations from independent experiments (*n* = 3). Mean values in the same row with different lowercase letters are significantly different (*p* < 0.05). ND: Not detected.

## Data Availability

Not applicable.

## Supplementary Materials

The following are available online at https://www.mdpi.com/article/10.3390/foods11030250/s1. Table S1: Ingredients of bread variants, information adapted from packaging of bread loafs (Gardenia); Table S2: Nutritional information of bread variants used, information adapted from packaging of bread loafs (Gardenia); Table S3: Qualitative observations on bread slurries of different concentrations; Table S4: Illustration of phase separation in fermented enriched white bread samples without and with additives (3% sweetener, 0.001% stabilizer) after 1 week of incubation at 30 °C; Table S5: Ethanol contents in unfermented bread slurry and fermented bread slurries at beginning and end of shelf life monitoring; Table S6: Selected volatile organic compounds (VOCs) in unfermented and fermented bread slurries at beginning and end of shelf life monitoring.
