# Supplementary material for: Biovalorization of Market Surplus Bread for Development of Probiotic-Fermented Potential Functional Beverages
Source: Foods. 2022 Jan 18;11(3):250. doi: 10.3390/foods11030250 (PMC8834041; doi:10.3390/foods11030250)
Supplement: Supplementary file 1 [file foods-11-00250-s001.zip › foods-1517639-supplementary.pdf]

Supplementary materials

Biovalorisation of market surplus bread for development of probiotic-fermented functional beverages

Thuy Linh Nguyen [1], Mingzhan Toh [1], Yuyun Lu [1,2,*], Sebastian Ku [3], Shao Quan Liu [1,2,*]

[1] Department of Food Science and Technology, Science Drive 2, Faculty of science, National University of Singapore, Singapore 117542, Singapore.

[2] National University of Singapore (Suzhou) Research Institute, 377 Lin Quan Street, Suzhou Industrial Park, Jiangsu 215123, China.

[3] Lesaffre Singapore Pte. Ltd., 23A Serangoon North Ave 5, #04-09, Singapore 554369, Singapore.

Corresponding Author

* Department of Food Science and Technology, Science Drive 2, National University of Singapore, Singapore 117543, Singapore. Tel.: 65-6516 2687. Fax: 65-6775-7895. E-mail: fstluy@nus.edu.sg (Yuyun Lu); fstlsq@nus.edu.sg (Shao Quan Liu)

Table S1. Ingredients of bread variants, information adapted from packaging of bread loafs (Gardenia).

EWB	FGWB	HCMB
Wheat flour (unbleached), sucrose, vegetable oil (palm), skimmed milk powder, common salt, dextrose, Baker's yeast, emulsifier, yeast nutrients (ammonium sulphate, sodium chloride, calcium sulphate), thiamine (vitamin B1), riboflavin (vitamin B2), niacin (vitamin B3), iron (hydrogen reduced iron), vitamin C, calcium propionate.	Wholemeal wheat flour with bran and wheat germ, wheat gluten, honey, vegetable oil (palm), skimmed milk powder, Baker's yeast, common salt, dextrose, emulsifiers, yeast nutrients (ammonium sulphate, sodium chloride, calcium sulphate), thiamine (vitamin B1), riboflavin (vitamin B2), niacin (vitamin B3), iron (hydrogen reduced iron), vitamin C, calcium propionate.	Wheat flour (unbleached), sucrose, skimmed milk powder, oat fibre, common salt, dextrose, vegetable oil (palm), calcium carbonate, Baker's yeast, emulsifier, inulin (oligosaccharide), calcium propionate, honey, thiamine (vitamin B1), riboflavin (vitamin B2), niacin (vitamin B3), iron (hydrogen reduced iron), flavouring, lutein, cholecalciferol (vitamin D3)

EWB: Enriched White Bread; FGWB: Fine Grain Wholemeal Bread; HCMB: Hi Calcium Milk Bread.

Table S2. Nutritional information of bread variants used, information adapted from packaging of bread loafs (Gardenia).

	EWB	FGWB	HCMB
Energy (kcal/100 g)	263	223	252
Protein (g/100 g)	9.9	12.1	10.3
Total fat (g/100 g)	1.9	2.7	1.5
Saturated fat (g/100 g)	0.9	1.2	0.8
Trans fat (g/100 g)	0.0	0.0	0.0
Cholesterol (mg/100 g)	0.0	0.0	0.0
Carbohydrate (g/100 g)	54.7	38.0	53.3
Total sugar	3.7	4.7	N/A
Dietary fibre (g/100 g)	2.5	5.3	3.0
Sodium (mg/100 g)	438	274	430
Vitamin B1 (mg/100 g)	0.77	0.5	0.7
Vitamin B2 (mg/100 g)	0.48	0.3	0.4
Vitamin B3 (mg/100 g)	5.06	3.1	5.1
Vitamin D3 (μg/100 g)	N/A	N/A	1.22
Lutein (μg/100 g)	N/A	N/A	80
Calcium (mg/100 g)	171.08	240.0	362
Iron (mg/100 g)	4.53	4.8	4.7

EWB: Enriched White Bread; FGWB: Fine Grain Wholemeal Bread; HCMB: Hi Calcium Milk Bread.
N/A = Not available (value not declared on packaging).

Table S3. Qualitative observations on bread slurries of different concentrations.

**Total bread solids (%)**	**1.25 – 5.0**	**10.0 – 25.0**
**Qualitative observations**	The bread slurries were flowable liquids suitable for beverage applications. Slight sedimentations were observed, the sediments could be dispersed by shaking.	The bread mixtures were solid-like, not flowable, and not suitable for beverage applications.
**Illustrating images**	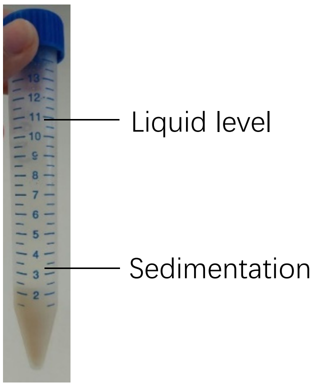	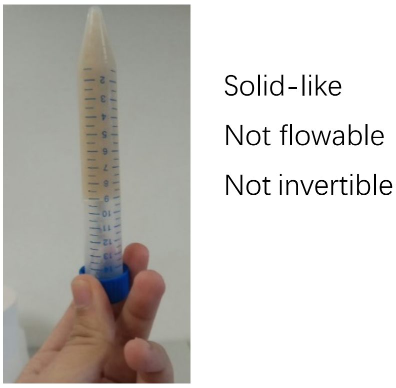

Table S4. Illustration of phase separation in fermented Enriched White Bread samples without and with additives (3% sweetener, 0.001% stabiliser) after 1 week of incubation at 30 °C.

**Without additives**	**With 3% sweetener + 0.001% stabiliser**
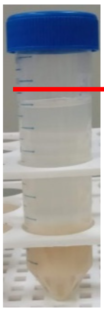	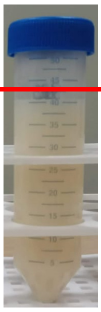

Table S5. Ethanol contents in unfermented bread slurry and fermented bread slurries at beginning and end of shelf life monitoring.

	Ethanol content (%)			
	Unfermented bread slurry	*L. rhamnosus* GG	*S. cerevisiae* CNCM I-3856	*L. rhamnosus* GG + *S. cerevisiae* CNCM I-3856
Week 0	0.09 ± 0.02^{a}	0.11 ± 0.02^{a}	0.30 ± 0.02^{b}	0.25 ± 0.01^{b}
Week 6 (5 °C)	-	0.09 ± 0.01^{a}	0.24 ± 0.03^{b}	0.22 ± 0.02^{b}
Week 6 (30 °C)	-	0.10 ± 0.02^{a}	0.27 ± 0.03^{c}	0.22 ± 0.02^{b}

Mean values in the same row with different lowercase letters are significantly different ($p < 0.05$).

Table S6. Selected volatile organic compounds (VOCs) in unfermented and fermented bread slurries at beginning and end of shelf life monitoring.

Compounds	LRI	GC-MS/FID peak area (× 10^6)									
		Unfermented bread slurry	*L. rhamnosus* GG			*S. cerevisiae* CNCM I-3856			*L. rhamnosus* GG + *S. cerevisiae* CNCM I-3856		
			Week 0	Week 6 (5 °C)	Week 6 (30 °C)	Week 0	Week 6 (5 °C)	Week 6 (30 °C)	Week 0	Week 6 (5 °C)	Week 6 (30 °C)
Acids											
Acetic acid	1450	0.28 ± 0.08^{a}	6.55 ± 1.82^{b}	5.90 ± 1.12^{b}	34.29 ± 12.71^{c}	2.21 ± 0.25^{b}	1.69 ± 0.48^{b}	5.49 ± 1.44^{b}	4.20 ± 1.70^{b}	5.48 ± 1.61^{b}	21.86 ± 5.42^{c}
Propionic acid	1532	4.54 ± 0.87^{a}	60.07 ± 18.87^{bc}	43.43 ± 12.94^{b}	97.52 ± 13.77^{c}	4.68 ± 0.52^{a}	7.61 ± 1.90^{a}	17.89 ± 7.34^{a}	71.02 ± 19.00^{bc}	84.38 ± 9.46^{c}	47.95 ± 10.84^{b}
Isobutyric acid	1561	0.28 ± 0.10^{a}	0.29 ± 0.02^{a}	0.32 ± 0.10^{a}	0.19 ± 0.06^{a}	0.90 ± 0.19^{b}	0.13 ± 0.02^{a}	0.14 ± 0.03^{a}	0.30 ± 0.06^{a}	0.32 ± 0.04^{a}	0.85 ± 0.14^{b}
Butyric acid	1622	ND	0.09 ± 0.01^{a}	0.15 ± 0.08^{a}	0.28 ± 0.07^{b}	ND	ND	ND	0.07 ± 0.02^{a}	0.08 ± 0.02^{a}	0.08 ± 0.02^{a}
Alcohols											
Ethanol	-	54.70 ± 8.33^{a}	63.26 ± 21.53^{a}	57.76 ± 7.43^{a}	61.39 ± 12.00^{a}	185.76 ± 91.85^{b}	277.80 ± 36.77^{b}	270.95 ± 47.42^{b}	194.84 ± 17.26^{b}	203.56 ± 23.17^{b}	176.16 ± 28.56^{b}
Isobutyl alcohol	1099	6.79 ± 0.56^{ab}	4.20 ± 1.05^{a}	2.55 ± 0.60^{a}	4.54 ± 0.29^{a}	16.64 ± 4.93^{bc}	18.30 ± 3.01^{c}	11.42 ± 1.96^{b}	6.07 ± 1.03^{ab}	5.32 ± 0.78^{a}	4.47 ± 0.62^{a}
Active amyl alcohol	1261	0.39 ± 0.15^{a}	0.55 ± 0.06^{a}	0.11 ± 0.02^{a}	4.81 ± 1.10^{b}	ND	ND	ND	0.41 ± 0.13^{a}	0.43 ± 0.08^{a}	0.43 ± 0.11^{a}
2-Ethyl-1-hexanol	1504	0.14 ± 0.02^{a}	0.33 ± 0.13^{b}	0.22 ± 0.02^{ab}	0.10 ± 0.01^{a}	ND	ND	ND	0.16 ± 0.03^{a}	0.14 ± 0.03^{a}	0.22 ± 0.03^{ab}
Furfuryl alcohol	1674	ND	ND	ND	ND	0.15 ± 0.02^{b}	0.07 ± 0.02^{a}	0.06 ± 0.00^{a}	ND	ND	ND
2-Phenethyl alcohol	1944	ND	ND	ND	ND	6.42 ± 2.77^{a}	13.36 ± 3.13^{b}	14.32 ± 4.67^{b}	ND	ND	19.75 ± 4.35^{b}
Ketones and Aldehydes											
Diacetyl	-	8.83 ± 0.35^{a}	13.12 ± 4.78^{a}	12.04 ± 0.68^{a}	12.67 ± 0.97^{a}	ND	ND	ND	ND	ND	ND
Hexanal	1076	2.80 ± 0.89^{a}	18.33 ± 4.14^{b}	15.37 ± 5.42^{b}	21.39 ± 6.58^{b}	ND	ND	ND	15.76 ± 4.41^{b}	17.36 ± 3.74^{b}	22.03 ± 5.44^{b}
2-Heptanone	1178	0.82 ± 0.20^{a}	0.97 ± 0.34^{a}	0.80 ± 0.39^{a}	1.02 ± 0.20^{a}	0.82 ± 0.22^{b}	0.47 ± 0.09^{a}	0.48 ± 0.06^{a}	0.76 ± 0.32^{a}	1.24 ± 0.35^{a}	0.97 ± 0.31^{a}
2-Octanone	1278	ND	0.07 ± 0.03^{a}	0.11 ± 0.02^{a}	0.08 ± 0.02^{a}	0.26 ± 0.07^{b}	0.13 ± 0.03^{a}	0.11 ± 0.01^{a}	0.04 ± 0.01^{a}	0.04 ± 0.01^{a}	0.03 ± 0.01^{a}
Acetoin	1291	3.14 ± 0.39^{b}	4.56 ± 1.58^{b}	3.11 ± 0.13^{b}	3.49 ± 0.69^{b}	0.35 ± 0.10^{a}	0.31 ± 0.10^{a}	0.37 ± 0.08^{a}	1.74 ± 0.35^{ab}	1.71 ± 0.34^{ab}	1.96 ± 0.32^{ab}
2-Octenal	1428	0.36 ± 0.10^{b}	0.23 ± 0.08^{ab}	0.44 ± 0.26^{ab}	0.30 ± 0.06^{b}	ND	ND	ND	0.24 ± 0.03^{ab}	0.20 ± 0.04^{a}	0.34 ± 0.07^{b}
Furfural	1471	0.11 ± 0.03^{b}	0.08 ± 0.01^{b}	0.08 ± 0.02^{b}	0.04 ± 0.01^{a}	ND	ND	ND	0.08 ± 0.00^{b}	0.10 ± 0.01^{b}	0.08 ± 0.03^{b}
Butyrolactone	1644	ND	ND	ND	ND	0.05 ± 0.01^{a}	0.34 ± 0.03^{b}	0.40 ± 0.06^{b}	ND	ND	ND
Esters											
Ethyl heptanoate	1319	ND	ND	ND	ND	ND	0.05 ± 0.02^{a}	0.06 ± 0.03^{a}	ND	ND	ND
Ethyl octanoate	1425	0.37 ± 0.05^{ab}	0.40 ± 0.16^{ab}	0.33 ± 0.12^{ab}	0.81 ± 0.26^{c}	0.66 ± 0.21^{bc}	0.48 ± 0.08^{b}	0.35 ± 0.13^{ab}	0.43 ± 0.06^{ab}	0.37 ± 0.03^{ab}	0.24 ± 0.05^{a}

Results expressed as mean ± standard deviations from independent experiments (n = 3). LRI: experimental linear retention index determined on a DB-FFAP column relative to C10-C40 alkane standard. ND = Not detected. Lowercase letters indicate significant differences ($p < 0.05$) in the same row.
